# Sooty mold deposition and leaf-level light attenuation beneath trees infested with *Lycorma delicatula*

**DOI:** 10.17912/micropub.biology.002211

**Published:** 2026-07-27

**Authors:** Jennifer Chandler

**Affiliations:** 1 Biology, West Chester University, West Chester, PA, United States

## Abstract

Sooty mold colonizes honeydew excreted by *Lycorma delicatula* during feeding, shading leaf surfaces. This study investigated the spatial “footprint” of sooty mold in understories of forests infested by *L. delicatula* , and how sooty mold coverage on leaves affects light transmission. Sooty mold accumulation decreased with increasing distance from the trunk to the canopy edge but was highly variable among trees. Light transmission decreased by 0.36% for every 1% increase in sooty mold coverage. A better understanding of the impacts of sooty mold in the understory is necessary, as understory woody regeneration shapes the long-term future of eastern forests.

**Figure 1. Spatial extent of sooty mold in the understories of forests infested by L. delicatula , and reductions in light transmission as a function of sooty mold coverage:** A) Deposition cards situated horizontally and extending outward at randomly-selected distances from the trunk of an *Ailanthus altissima* tree. This image shows only the first three of approximately 13 cards that spanned the entire distance from the trunk of the tree to the edge of the tree canopy B) Examples of two deposition cards covered with varying amounts of sooty mold C) Depiction of how distances from the trunk of a tree were relativized to percent total distance from trunk to canopy edge D) Percent area of deposition cards covered by sooty mold as a function of the relative distance of each card from the trunk to the canopy edge. Different colored markers represent data collected under a single tree, as tree was incorporated as a random variable in the analysis E) Example of leaves used for percent change in PAR transmission. Note the areas where sooty mold was removed so that PAR minus sooty mold could be remeasured F) Percent change in light transmission through a leaf as a function of the percent cover of sooty mold

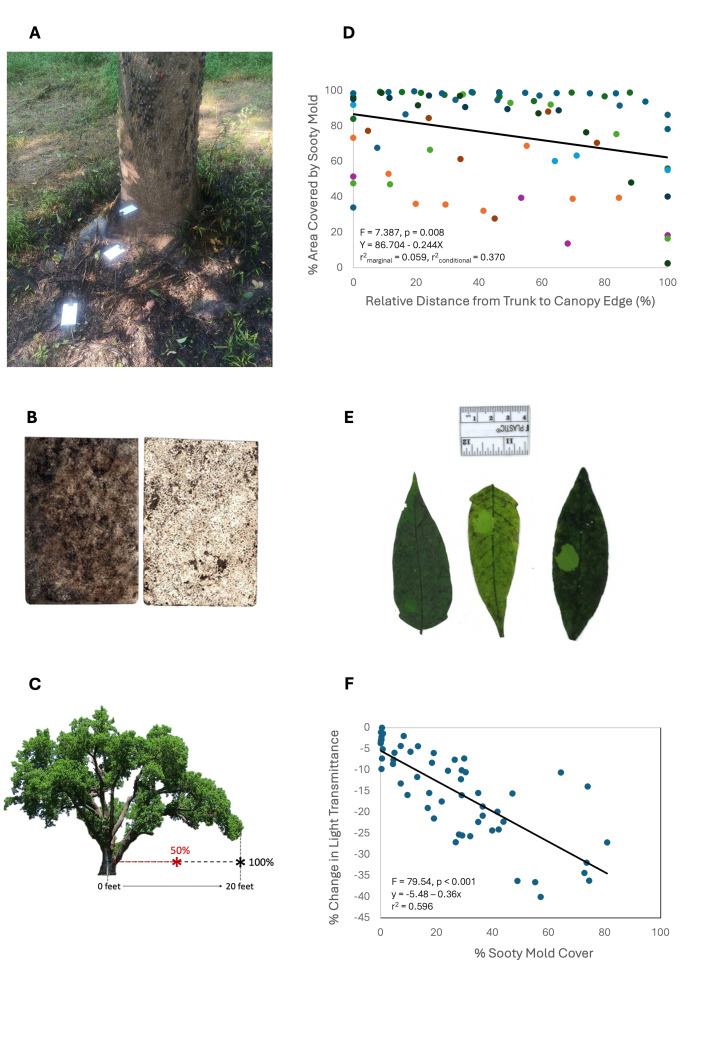



## Description

*Lycorma delicatula* (White), commonly known as spotted lanternfly, was first observed in Berks County Pennsylvania, U.S.A., in 2014 and quickly became an invasive insect of concern in the mid-Atlantic region (Barringer et al., 2015; Lee et al., 2019; Urban et al., 2021). Since its introduction, *L. delicatula* has spread to 18 additional states, raising concern for native species throughout the eastern United States (Nixon et al., 2026). While the invasive *Ailanthus altissima* (Mill.) Swingle is the preferred host of *L. delicatula* , the pest is polyphagous, feeding on a wide variety of over 70 additional species (Dara et al., 2015; Lee et al., 2019; Barringer and Ciafré, 2020; Urban et al., 2021; McPherson et al., 2022). Like other sap sucking insects, *L. delicatula* use piercing-sucking mouthparts to access the host’s phloem sap to feed, and simultaneously excretes sugary honeydew (Dara et al., 2015; Hao et al., 2016). Research suggests that feeding by *L. delicatula* can cause oozing, wilting, death of branches and limbs, alterations to plant physiology and growth, and in some cases, whole tree death (Dara et al., 2015; Lavely et al., 2022; Hoover et al., 2023; Zhang et al., 2023; personal observations).

While much research has focused on understanding the variety of plant species on which *L. delicatula* feeds (Dara et al., 2015; Lee et al., 2019; Barringer and Ciafré, 2020; Urban et al., 2021; McPherson et al., 2022) and the *direct* impact that feeding has on plant health (Dara et al., 2015; Lavely et al., 2022; Hoover et al., 2023; Zhang et al., 2023), less has focused on the *indirect* effects that feeding by *L. delicatula* has on forest systems. As mentioned above, honeydew is excreted by *L. delicatula* during feeding. The sugary honeydew that falls and coats branches and leaves of lower strata is often colonized by dark pigmented fungi collectively known as sooty mold, particularly in areas with high rates of *L. delicatula* infestation (Lee et al., 2009; Dara et al., 2015; Hao et.al., 2016; Lee et al., 2019; Urban and Leach, 2023). Sooty mold does not penetrate the epidermis, and thus does not cause structural damage to the leaf (Lemos Filho and Paiva, 2006). However, sooty mold does shade impacted leaves, leading to potential reductions in photosynthesis, and in extreme cases, withering and plant death (Lee et al., 2009; Insausti et al., 2015; Urban and Leach, 2023). Urban and Leach (2023) suggest that more research on sooty mold deposition in forested areas is needed to better understand the full range of *L. delicatula* impact. One starting point for this work is to characterize the spatial extent of sooty mold in forests infested by *L. delicatula* , and to assess its shading potential on understory plants, given the vulnerability of this stratum to sooty mold colonization and the consequent risk to advanced regeneration.

The purpose of this work was two-fold. First, I wanted to understand how sooty mold coverage in the understory varies under the canopies of trees infested by *L. delicatula* . Much of the feeding occurs on the main trunk of a tree, especially in late summer when adults are predominant, though later instars and adults are often observed feeding on lateral branches, as well. As such, I predicted that sooty mold cover in the understory would be highest adjacent to the trunk, and would rapidly decrease as distance from the trunk increased. To test this, I placed white deposition cards at varying distances from the trunk to the edge of the canopy of ten infested trees spanning two states ( Fig. 1A ). Cards were left in the field for several weeks in late summer to allow sooty mold to colonize ( Fig. 1B ), after which time I quantified the sooty mold coverage on each card. I used a linear mixed-effect model to explore how the area covered by sooty mold varies in relation to the relative distance from the trunk (Fig 1C), including tree as a random factor in the model, since samples taken under the same trees were not independent. Distances were relativized as a percentage of the distance from the trunk of the tree to the canopy edge to account for the fact the trees in the study varied greatly in size ( Fig. 1C ). My results suggest that sooty mold coverage decreased with increasing relative distance from the trunk ( Fig. 1D ; F = 7.387, p = 0.008). Relative distance, coupled with tree ID, explained a substantial portion of variance in mold coverage (r ^2^_conditional_ = 0.370), while the effect of relative distance from the trunk alone was more modest (r ^2^_marginal_ = 0.059). Nevertheless, independent of tree-to-tree differences, sooty mold accumulation was consistently concentrated closer to the stem and declined toward the canopy edge. In all, accumulation patterns are highly variable among trees (Fig 1D), with some trees exhibiting heavy sooty mold cover close to the trunk that slowly tapers off toward the edge of the canopy, and other trees maintaining a similar level of sooty mold coverage from the trunk to the edge of the canopy.

The second objective of this work was to understand how sooty mold coverage on the adaxial side of leaves affects light transmission. I predicted that light transmission would decrease rapidly with increasing sooty mold coverage. To test this, I measured the change in the percent light transmitted through a leaf when sooty mold was present on the adaxial surface of the leaf compared to when the sooty mold was removed (Fig 1E). I then regressed the percent change in light transmission over the percent area of sooty mold cover that was present on the leaf. The change in the percent of light transmitted through the leaf decreased as the percent of sooty mold that covered the adaxial side of the leaf increased ( Fig. 1F ; F = 79.54, p < 0.001, r ^2^ = 0.596). Light transmission decreased by approximately a third of a percent (0.36%) for every 1% increase in sooty mold.

It is clear from this work that the spatial extent, or “footprint” of sooty mold deposition in infested forests is variable and highly nuanced. While my work indicates that sooty mold accumulation decreases steadily as the distance from the trunk to the edge of the tree’s canopy increases, the observed pattern is weak, and sooty mold accumulation in lower strata is likely driven more by branching patterns of individual trees. More extensive work is needed to investigate these patterns, and should include substantial replication both within and among tree species, as species-specific limb architecture will impact sooty mold footprints in the understory. Nevertheless, the relatively strong relationship between light transmission and sooty mold coverage suggests that sooty mold colonization results in high levels of cover-dependent light attenuation, which could substantially impact a plant’s ability to photosynthesize. Since the future of eastern forests is largely dependent on the advanced regeneration that currently exists in the forest understory, an understanding of the physiological and growth impacts of sooty mold colonization in this stratum is critical, and future studies should investigate the response of woody understory species to sooty mold shading.

## Methods

Question 1 – *Sooty mold cover as a function of relative distance from infested tree*

In summer 2020, five *A. altissima* infested with *L. delicatula* were located in Frederick County, VA and five in Chester County, PA. Canopy diameter among the trees ranged from approximately 5.2m – 12.2m. Field techniques based on work described in an ArborJet gray paper (Doccola et al., date unknown) were employed, as follows: white plastic deposition cards (5.08cm. x 7.62cm.) were situated horizontally one meter above the forest floor at random distances from the main trunk of the tree in directions that: 1) increased the likelihood of honeydew interception by taking the form of the tree into consideration, and 2) avoided confounding canopies (Fig 1A). Multiple deposition cards were deployed per tree, with the first card at each tree always situated adjacent to the trunk and the last card placed at the edge of the tree’s canopy. Additional cards were located at random distances between these two cards. Deposition cards were left in the field for several weeks in late summer to accumulate sooty mold, after which time they were collected and photographed (Fig 1B). Percent cover of sooty mold on each deposition card was calculated using ImageJ. The distance each card was placed from the trunk of the tree was relativized as the percent distance from the trunk to the edge of the tree’s canopy for all eighty of the deployed deposition cards ( Fig. 1C ). Relativization was critical, because mold accumulation 2m from the trunk of a very small tree, for example, would be inherently different than accumulation 2m from the trunk of a very large tree due to differences in the location of the sample within the canopy shadow (drip line on the smaller tree vs. dense canopy in the larger tree). To evaluate the relationship between sooty mold coverage and relative distance from the trunk, I used a linear mixed-effects model (REML) with relative distance as a fixed effect, and tree ID as a random effect to account for non-independence among samples taken below the same tree. Data were analyzed using SPSS (v.29, IBM).

Question 2 – *Change in light transmission as a function of percent sooty mold coverage*

Leaves from three woody species ( *A. altissima* , *Elaeagnus umbellate* Thunb., and *Carya* ssp.) were selected haphazardly based on their availability in the understory of *L. delicatula* infested sites, ensuring that they spanned a range of sooty mold coverage (Fig 1E). Using a standardized relative measure of percent change in Photosynthetically Active Radiation (PAR) transmission, I quantified the change in PAR transmitted through each leaf when sooty mold was present compared to when sooty mold was removed. Standardization was necessary since I was measuring transmission *through* a leaf, and leaf thickness varied both among and within species. A stationary light source was positioned facing downward 2cm above a stationary quantum sensor. One at a time, leaves were situated flush over the sensor, PAR transmission through the leaf and any attached sooty mold was recorded, and the location on the leaf where the measurement was taken was marked. Images of each leaf were taken, and the percent cover of sooty mold was evaluated using ImageJ software. Surface sooty mold was gently removed from within the marked area of each leaf using a damp cotton swab ( Fig. 1E ). Leaves were processed identically regardless of whether surface sooty mold was observed. Leaves were placed back on the sensor in the identical configuration as before, and PAR transmission was re-measured. The percent change in transmission was calculated for each leaf as:

% change transmission = 100 * ((PAR _PlusSootyMold_ - PAR _MinusSootyMold_ )/(PAR _MinusSootyMold_ ))

I regressed percent change in light transmission over percent area of sooty mold cover to determine how percent area of sooty mold on the adaxial side of a leaf affects percent PAR transmission. Data were analyzed using SPSS (v.29, IBM).
